# Serum Cystatin C and Coronavirus Disease 2019: A Potential Inflammatory Biomarker in Predicting Critical Illness and Mortality for Adult Patients

**DOI:** 10.1155/2020/3764515

**Published:** 2020-10-08

**Authors:** Dan Chen, Wenwu Sun, Jia Li, Bohua Wei, Wei Liu, Xiaopin Wang, Fan Song, Liangkai Chen, Junhui Yang, Li Yu

**Affiliations:** ^1^Intensive Care Unit, The Central Hospital of Wuhan, Tongji Medical College, Huazhong University of Science and Technology, Wuhan, China; ^2^Department of Health Technology and Informatics, The Hong Kong Polytechnic University, Hung Hom, Kowloon, Hong Kong; ^3^Department of Nutrition and Food Hygiene, Hubei Key Laboratory of Food Nutrition and Safety, Ministry of Education Key Lab of Environment and Health, School of Public Health, Tongji Medical College, Huazhong University of Science and Technology, Wuhan, China

## Abstract

This study aimed at determining the relationship between baseline cystatin C levels and coronavirus disease 2019 (COVID-19) and investigating the potential prognostic value of serum cystatin C in adult patients with COVID-19. 481 patients with COVID-19 were consecutively included in this study from January 2, 2020, and followed up to April 15, 2020. All clinical and laboratory data of COVID-19 patients with definite outcomes were reviewed. For every measure, COVID-19 patients were grouped into quartiles according to the baseline levels of serum cystatin C. The highest cystatin C level was significantly related to more severe inflammatory conditions, worse organ dysfunction, and worse outcomes among patients with COVID-19 (*P* values < 0.05). In the adjusted logistic regression analyses, the highest cystatin C level and ln-transformed cystatin C levels were independently associated with the risks of developing critically ill COVID-19 and all-cause death either in overall patients or in patients without chronic kidney disease (*P* values < 0.05). As a potential inflammatory marker, increasing baseline levels of serum cystatin C might independently predict adverse outcomes for COVID-19 patients. Serum cystatin C could be routinely monitored during hospitalization, which showed clinical importance in prognosticating for adult patients with COVID-19.

## 1. Introduction

At the end of 2019, serial clinical cases of unknown etiological pneumonia were reported in Wuhan, a provincial capital city of Hubei, China [1, 2]. Within weeks of the early investigations, the pathogenic organism was identified as a novel coronavirus (2019-nCoV), subsequently named as severe acute respiratory syndrome coronavirus 2 (SARS-CoV-2) [3]. This formidable SARS-CoV-2 is similar to other pathogens in the coronavirus family, with rapid transmission from human to human [4]. A public health alert is now upgraded to an international emergency owning to the outbreak and pandemic of coronavirus disease 2019 (COVID-19) caused by SARS-CoV-2, imposing a huge burden all over the world [5].

COVID-19 has been revealed a wide clinical range from asymptomatic infection to critical pneumonia with acute respiratory distress syndrome (ARDS) [6–8]; even so, the prevalence of multiple organ dysfunction and the mortality were still high among patients with COVID-19 [9, 10]. The exacerbation of COVID-19 was largely accompanied by violent cytokine storm due to overactive inflammation and immune response [11]. Cystatin C is a cysteine protease inhibitor secreted from human cells to blood circulation and directly interacts with human coronaviruses [12]. Apart from the indication of renal function [13], serum cystatin C originated from human alveolar macrophages might also associate with lung inflammation and respiratory dysfunction [14, 15]. Furthermore, cystatin C was suggested as an independent predictive factor of high mortality among the elderly population [16, 17]. As yet, little was known of the relationship between circulating cystatin C levels and disease severity or all-cause death in COVID-19.

In this hospital-based cohort study, all laboratory-confirmed patients with COVID-19 were recruited from a single center, and their clinical data were retrospectively analyzed to determine the relevance of serum cystatin C levels to the risks of critical illness and subsequent mortality among COVID-19 patients.

## 2. Materials and Methods

### 2.1. Study Subjects

In this retrospective cohort study, 481 consecutive adult subjects were confirmed as COVID-19, when admitted to the Central Hospital of Wuhan from January 2, 2020, and followed up to April 15, 2020. The diagnosis of COVID-19 was based on the World Health Organization interim guidance [18]. Throat swab specimens at admission were gathered from any suspected patients. Real-time reverse transcription-polymerase chain reaction was utilized to confirm SARS-CoV-2 infection in accordance with the manufacturer's protocol (Beijing Genomics Institution and Geneodx biotechnology Co., Ltd.). The verification of virus clearance prior to hospital discharge was required for each confirmed case via repeated tests of SARS-CoV-2. Only SARS-CoV-2-infected patients identified by throat swab samples were included in the current study. Any patient was excluded with the following conditions: a history of craniocerebral surgery or carcinomas; any recent life-threatening organic disease within 3 months; died on admission; and transferred to other hospitals. All data were anonymous, and oral informed consents were obtained from all participants due to the emergency of COVID-19 outbreaks. The institutional Ethics Committees approved this study, which followed the Declaration of Helsinki.

### 2.2. Data Collection

A trained physician team reviewed all clinical parameters from case report forms, nursing records, laboratory results, and radiological features. Patient electronic medical records provided in-hospital information on demographic characteristics, comorbidities, clinical manifestations, laboratory findings, therapy, and outcomes. Clinical laboratory markers, such as arterial blood lactate, PaO_2_/FiO_2_ ratio, white blood cell count, lymphocyte count, neutrophil/lymphocyte ratio, platelet count, hemoglobin, C-reactive protein, procalcitonin, cystatin C, blood urea nitrogen, creatinine, total bilirubin, alanine aminotransferase, aspartate aminotransferase, fibrinogen, D-dimer, lactate dehydrogenase, and creatine kinase, were measured and collected at admission. As stated by the testing Project Manual and reagent directions of the hospital, the normal reference interval of cystatin C was 0.6-1.55 mg/L. Acute Physiology and Chronic Health Evaluation II (APACHE II) and Sequential Organ Failure Assessment (SOFA) scores were summarized within 24 h after admission. ARDS was diagnosed in line with the Berlin definition [19]. The severity of COVID-19 was classified according to the Handbook of COVID-19 Prevention and Treatment [20]. Other data on therapeutic management were also obtained during hospitalization. Patient outcomes were monitored up to their death or discharge.

### 2.3. Statistical Analysis

Baseline continuous variables were shown as median (interquartile range, IQR), while categorical variables as *n* (%). To present and compare the baseline characteristics, all patients were divided into quartiles according to the levels of serum cystatin C. For continuous variables, a generalized liner regression model was applied to obtaining the *P* value of the trend test. For categorical variables, chi-squared test or Fisher's exact test was utilized. Differences in cystatin C levels between groups were determined by the Mann–Whitney *U* test. To estimate the association of baseline cystatin C with the risks of critical illness and all-cause mortality, logistic regression models were used in terms of the following confounding factors: age, sex, chronic pulmonary disease, hypertension, diabetes, cardiovascular disease, cerebrovascular disease, and chronic kidney disease (CKD). Both cystatin C and blood creatinine may represent renal function, and patients with acute kidney injury (AKI) are more likely to develop critical illness or death. Besides, a high APACHE II score may reflect renal organ failure and associate with high mortality in critically ill patients. For these reasons, blood creatinine levels and APACHE II scores were also considered in the regression models. The Kaplan-Meier methods were used for survival curve plotting. The R software (The R Foundation, http://www.r-project.org, version 3.6.1) and EmpowerStats (http://www.empowerstats.com, X&Y Solutions, Inc., Boston, MA) were utilized for analyzing all statistics. A two-sided level of *P* < 0.05 was defined as statistically significant.

## 3. Results

### 3.1. Clinical Characteristics Associated with Serum Cystatin C Levels

The baseline clinical parameters of all 481 subjects with COVID-19 were summarized according to the quartile levels of serum cystatin C in Table 1. Compared to the lower cystatin C levels (<1.36 mg/L), the highest cystatin C level (≥1.36 mg/L) was associated with older age, higher prevalence among male patients, and higher incidence of the coexistent conditions, such as chronic pulmonary disease, hypertension, diabetes, cardiovascular disease, cerebrovascular disease, and CKD (all *P* values for trend < 0.05, Table 1). The highest cystatin C level was also found in significant association with increased lactate levels, decreased PaO_2_:FiO_2_ ratio, and higher incidence of ARDS (all *P* values for trend < 0.05, Table 1). The occurrence of critically ill COVID-19 was more prevalent in the highest cystatin C group, with APACHE II and SOFA scores ascending (all *P* values for trend < 0.05, Table 1).

The highest cystatin C level was significantly associated with higher admission levels of white blood cell count, C-reactive protein, and procalcitonin, as well as greater neutrophil/lymphocyte ratio (all *P* values for trend < 0.05, Table 1). Besides, statistical differences were detected in the baseline levels of blood urea nitrogen, creatinine, total bilirubin, alanine aminotransferase, aspartate aminotransferase, D-dimer, and lactate dehydrogenase (all *P* values for trend < 0.05, Table 1). Except for total bilirubin and alanine aminotransferase, other biomarkers had significantly higher levels in the highest cystatin C group.

Patients in the highest cystatin C group were more likely to be treated with Oseltamivir and noninvasive ventilation, but less likely to receive glucocorticoid therapy (all *P* values for trend < 0.05, Table 1). Longer in-hospital stays for survivors were observed in the highest cystatin C group, while the mortality rate in this group (33%) was significantly higher (all *P* values for trend < 0.05, Table 1).

Similarly, there were significant differences in the levels of cystatin C between different subgroups, as shown in Table 2. A higher admission level of serum cystatin C was seen in males, elderly patients (over 65 years old), patients with each comorbidity, and nonsurvivors (all *P* values < 0.05, Table 2). In addition, increased baseline levels of serum cystatin C were significantly related to higher levels of blood creatinine, higher APACHE II scores, and critical illness in COVID-19 (all *P* values < 0.05, Table 2).

### 3.2. Association of Cystatin C with Mortality and Critical Illness in all Subjects

Several potential risk factors of disease severity and all-cause death for COVID-19 were adjusted in the logistic regression models, including age, sex, chronic pulmonary disease, hypertension, diabetes, cardiovascular disease, cerebrovascular disease, CKD, blood creatinine levels, and APACHE II scores. As presented in Table 3, either the highest cystatin C level (≥1.36 mg/L) or ln-transformed cystatin C levels were independently related to the elevated risks of developing critical illness and mortality in all COVID-19 patients, after adjusting for the above confounders (all *P* values < 0.05).

**Figure 1:**
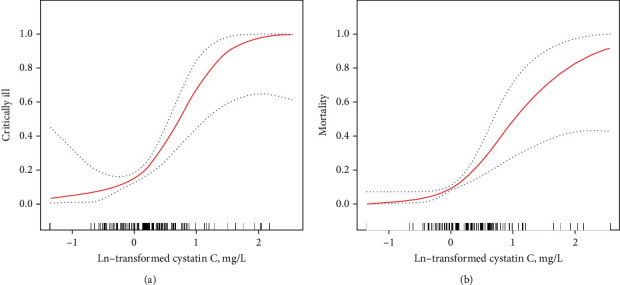
The nonlinear relationship between baseline ln-transformed cystatin C levels and the prevalence of critical illness (a) and mortality (b) in all COVID-19 patients. The generalized additive model was used for presenting the smoothing splines, adjusted for age, sex, blood creatinine levels, APACHE II scores, and coexistent conditions (such as chronic pulmonary disease, hypertension, diabetes, cardiovascular disease, cerebrovascular disease, and chronic kidney disease). The red lines suggested the risks of critical illness (a) and mortality (b), respectively. The blue dot lines suggested 95% confidence intervals.

**Figure 2:**
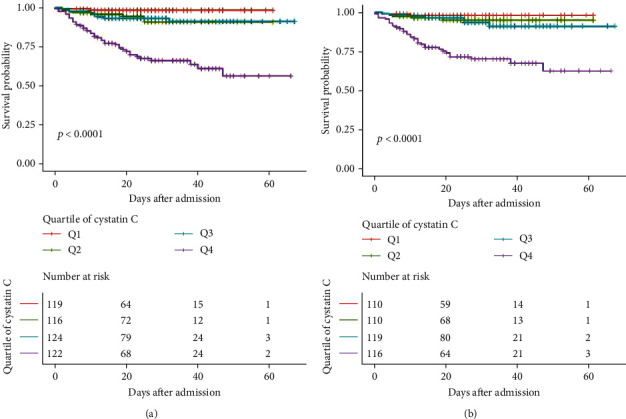
The Kaplan-Meier curves categorized by the quartiles of baseline serum cystatin C levels. (a) In overall COVID-19 patients, serum cystatin C on admission was grouped into the levels of <0.90 mg/L (Q1), 0.90-1.08 mg/L (Q2), 1.08-1.36 mg/L (Q3), and ≥1.36 mg/L (Q4). (b) In COVID-19 patients without chronic kidney disease, serum cystatin C on admission was grouped into the levels of <0.89 mg/L (Q1), 0.89-1.06 mg/L (Q2), 1.06-1.31 mg/L (Q3), and ≥1.31 mg/L (Q4).

The nonlinear association of ln-transformed cystatin C levels with critical illness and mortality in COVID-19 was then visually described in Figure 1. The prevalence of critically ill COVID-19 ascended with increasing ln-transformed cystatin C levels, and a gradual tendency of the gentle curve was observed around ln-transformed cystatin C levels of more than 1.50 mg/L (Figure 1(a)). Admission levels of ln-transformed cystatin C were also positively associated with the mortality among COVID-19 patients, which indicated that higher ln-transformed cystatin C levels might imply higher death rates during hospitalization of patients with COVID-19 (Figure 1(b)). Subsequent analysis via the Kaplan-Meier methods revealed that overall COVID-19 patients with a baseline cystatin C level ≥ 1.36 mg/L had significantly worse outcomes, compared to those with cystatin C levels < 1.36 mg/L (*P* value < 0.0001, Figure 2(a)).

### 3.3. Association of Cystatin C with Mortality and Critical Illness in Subjects without CKD

To minimize the impact of coexisting CKD on the progression of COVID-19, CKD patients were excluded in the following analyses. 455 subjects without CKD were then categorized by the quartiles of admission serum cystatin C levels (<0.89 mg/L; 0.89-1.06 mg/L; 1.06-1.31 mg/L; and ≥1.31 mg/L). The logistic regression models in Table 4 adjusted for age, sex, chronic pulmonary disease, hypertension, diabetes, cardiovascular disease, cerebrovascular disease, blood creatinine levels, and APACHE II scores. The highest cystatin C level (≥1.31 mg/L) was an independent risk factor for critically ill COVID-19 and all-cause death in patients without CKD, after adjustment for the underlying confounders (all *P* values < 0.05, Table 4). ln-transformed cystatin C levels were also independently associated with the risks for critical illness and mortality among COVID-19 patients without CKD (all *P* values < 0.05, Table 4). Additionally, the Kaplan-Meier analysis further suggested that the worst outcome was observed in the baseline cystatin C levels ≥ 1.31 mg/L (*P* value < 0.0001, Figure 2(b)).

## 4. Discussion

To the best of our knowledge, this study is the first to report the relationship between baseline levels of cystatin C and COVID-19 outcomes, and to highlight the prognostic values of monitoring serum cystatin C on hospitalized patients with SARS-CoV-2 infection. Firstly, clinical features of COVID-19 patients were compared under the quartile classification of baseline cystatin C levels (less than 0.90, 0.90 to 1.08, 1.08 to 1.36, and more than 1.36 mg/L, respectively). We found that the highest cystatin C level on admission was strongly associated with more severe inflammatory status, worse organ dysfunction, and worse outcomes among patients with COVID-19. Secondly, the highest cystatin C level and ln-transformed cystatin C levels were identified in the adjusted analyses as independent risk factors for critical illness and death from all causes in overall patients with COVID-19. Thirdly, even after CKD patients were excluded in the logistic regression models, the highest cystatin C level and ln-transformed cystatin C levels were still independently associated with the risks of developing critically ill COVID-19 and subsequent mortality. Therefore, circulating cystatin C could serve as a potential inflammatory target for preventing COVID-19 from the likely progression of critical illness and in-hospital death, not just representing kidney function.

In the pathological process of infection and inflammation, the crucial functions of cystatin C need to be emphasized [12]. After abundant secretion into various human body fluids, cystatin C extracellularly suppresses the activation of cysteine proteases that are utilized by invading pathogens into the host [21–23]. Accordingly, recombinant human cystatin C was reported to play an inhibitory role during the growth period of human coronaviruses [24]. The interference of cystatin family with replicating human coronavirus was also found in the host lung cells [25]. Besides, cytokines from inflammatory cells can modulate the expressions of cystatin C gene [26], while cystatin C will, in turn, fundamentally influence immune regulation to maintain homeostasis in the host immune system [12].

Cystatin C is easily detectable in the biological fluids and acts as a reliable marker, independently of age, sex, and muscle mass [27–29]. Previous studies not only confirmed the role of cystatin C in indicating organ dysfunction but also clarified that serum cystatin C levels might associate with inflammatory phase and adverse outcomes in various diseases [30–33]. These findings accorded with our observations of cystatin C levels and COVID-19. Of note, a multicenter cohort study of pediatric subjects infected by human immunodeficiency viruses (HIV) demonstrated that cystatin C levels were strongly related to kidney function and inflammatory state [34]. Positive relevance of elevating cystatin C levels to plasma HIV RNA status was also observed in adult patients with HIV infection whose antiretroviral therapy was randomly interrupted [35]. This underlying interaction between circulating cystatin C and viral infection may provide insight into our understanding of pathophysiological events in COVID-19.

A serious deterioration in COVID-19 was mainly characterized by the evolution of ARDS [36]. In the inflammatory course of ARDS due to SARS-CoV-2, lymphocytes infiltrated into lung tissues, while the host immune system was excessively activated and aggravated tissue injury [36]. In part, our findings might account for this phenomenon. First, the incidence rates of ARDS and critically ill COVID-19 were obviously higher among patients with the highest cystatin C level, who were also accompanied by elevated lactate levels, but a reduced PaO_2_:FiO_2_ ratio. Second, the highest cystatin C level was significantly related to the increased levels of inflammatory indexes, including white blood cell count, C-reactive protein, procalcitonin, neutrophil/lymphocyte ratio, and D-dimer. In addition to acute lung tissue damage, renal dysfunction was reported in COVID-19 patients as well [7, 9]. If kidney disease occurred at admission or AKI progressed during hospitalization, COVID-19-related death rate was ascended dramatically [37]. In the current study, we found positive association of a higher cystatin C level with increased values of blood creatinine and APACHE II score. However, the logistic regression models adjusting for these confounding factors further revealed that elevated levels of serum cystatin C were independently associated with the risks for critical illness and death in COVID-19. We thus reckon that circulating cystatin C may function as a potential biomarker of systemic inflammation during the exacerbation of COVID-19, rather than only reflecting renal function in patients infected by SARS-CoV-2.

Generally, the process of COVID-19 involved the overactivation of inflammatory and immune responses in severely ill cases, which eventually resulted in the high mortality for SARS-CoV-2-infected patients [38, 39]. Our results are meaningful. The highest admission level of serum cystatin C was strongly associated with more severe inflammation, worse organ dysfunction, and worse outcomes in COVID-19. Moreover, increasing baseline levels of cystatin C were independently related to the risks of occurring critical illness and in-hospital death either in overall COVID-19 patients or in COVID-19 patients without CKD. In other words, higher cystatin C levels on admission might predict significantly worse outcomes for adult patients with COVID-19. Yet, the underlying mechanism is still unclear. Our observations should be further verified with more basic and clinical studies.

Limitations should be considered in this study. Firstly, the causal relationship between serum cystatin C and COVID-19 outcomes could not be concluded, due to the nature of an observational research. Secondly, the likelihood of other residual or unnoticeable confounders could not be excluded. Thirdly, we did not investigate whether the dynamic changes of cystatin C were associated with long-term outcomes, because of partial missing data on follow-up.

## 5. Conclusions

In this hospital-based study of 481 adult patients confirmed as COVID-19, we revealed that baseline serum cystatin C might serve as a potential inflammatory biomarker, independently predicting poor outcomes for SARS-CoV-2 infection. Monitoring cystatin C during in-hospital stays may thus be of clinical importance in the prognosis for adult patients with COVID-19.

## Figures and Tables

**Table 1:** Baseline clinical features of patients with COVID-19, according to quartiles of cystatin C levels.

Characteristic	Quartile of serum cystatin C, mg/L				*P* trend
	Quartile 1 (*n* = 119) <0.90	Quartile 2 (*n* = 116) 0.90-1.08	Quartile 3 (*n* = 124) 1.08-1.36	Quartile 4 (*n* = 122) ≥1.36	
Age, median (IQR), y	45 (34-59)	53 (37-64)	62 (49-71)	68 (59-77)	<0.001
Male, no. (%)	37 (31%)	43 (37%)	69 (56%)	78 (64%)	<0.001
Comorbidities, no. (%)					
Chronic pulmonary disease	6 (5%)	4 (3%)	10 (8%)	17 (14%)	0.01
Hypertension	24 (20%)	39 (34%)	41 (33%)	80 (66%)	<0.001
Diabetes	15 (13%)	17 (15%)	15 (12%)	44 (36%)	<0.001
Cardiovascular disease	12 (10%)	8 (7%)	16 (13%)	25 (20%)	0.01
Cerebrovascular disease	1 (1%)	5 (4%)	7 (6%)	29 (24%)	<0.001
Chronic kidney disease	0	0	1 (1%)	25 (20%)	<0.001
Blood gas and severity					
PaO_2_:FiO_2_, median (IQR)	420 (296-521)	385 (271-520)	322 (240-485)	274 (180-406)	<0.001
Lactate, mmol/L, median (IQR)	1.2 (0.8-1.8)	1.2 (0.8-1.9)	1.4 (0.9-2.0)	1.5 (0.9-2.3)	0.02
ARDS, no. (%)	30 (25%)	38 (33%)	58 (47%)	69 (57%)	<0.001
Severity					
General, no. (%)	82 (69%)	75 (65%)	56 (45%)	32 (26%)	
Severe, no. (%)	27 (23%)	29 (25%)	48 (39%)	28 (23%)	
Critically ill, no. (%)	10 (8%)	12 (10%)	20 (16%)	62 (51%)	<0.001
SOFA score, median (IQR)	1 (0-6)	1 (0-7)	2 (0-7)	3 (0-10)	<0.001
APACHE II score, median (IQR)	2 (0-16)	2 (0-20)	3 (0-15)	7 (0-21)	<0.001
Admission laboratory markers, median (IQR)					
White blood cell count, ×10^9^/L	4.6 (3.5-5.7)	4.8 (4.1-6.0)	5.0 (4.0-6.6)	5.4 (4.1-7.7)	<0.001
Lymphocyte count, ×10^9^/L	1.1 (0.7-1.4)	1.1 (0.8-1.4)	1.0 (0.7-1.3)	0.8 (0.5-1.2)	0.11
Neutrophil/lymphocyte ratio	2.6 (1.7-4.1)	3.1 (1.7-5.2)	3.6 (2.2-6.2)	4.9 (2.6-9.5)	<0.001
Platelet count, ×109/L	175 (147-228)	186 (151-245)	186 (139-232)	162 (118-224)	0.75
Hemoglobin, g/L	128 (120-140)	127 (118-138)	130 (119-142)	124 (110-136)	0.11
C-reactive protein, mg/dL	1.1 (0.3-3.2)	1.8 (0.5-4.6)	2.2 (0.7-5.5)	3.9 (1.4-7.1)	<0.001
Procalcitonin, ng/mL	0.05 (0.04-0.06)	0.05 (0.04-0.08)	0.06 (0.04-0.10)	0.12 (0.05-0.31)	<0.001
Blood urea nitrogen, mmol/L	3.6 (3.0-4.8)	3.7 (3.1-4.3)	4.3 (3.5-5.3)	6.3 (4.6-8.9)	<0.001
Creatinine, *μ*mol/L	56 (48-71)	57 (49-70)	70 (53-80)	88 (71-114)	<0.001
Total bilirubin, mmol/L	8.2 (6.2-10.5)	8.4 (6.9-11.7)	9.9 (7.5-12.8)	8.8 (6.0-11.9)	0.008
Alanine aminotransferase, U/L	18 (13-28)	22 (13-32)	25 (15-43)	19 (14-32)	0.04
Aspartate aminotransferase, U/L	20 (17-29)	23 (18-34)	25 (19-41)	26 (18-37)	0.005
Fibrinogen, g/L	2.8 (2.4-3.2)	3.0 (2.5-3.4)	3.1 (2.6-3.5)	3.3 (2.6-3.8)	0.07
D-dimer, mg/L	0.40 (0.23-0.90)	0.49 (0.28-1.03)	0.69 (0.32-2.24)	0.91 (0.50-2.39)	0.03
Lactate dehydrogenase, U/L	182 (140-232)	192 (152-258)	207 (173-256)	218 (171-296)	<0.001
Creatine kinase, U/L	79 (52-147)	72 (45-119)	77 (53-124)	88 (60-153)	0.90
Creatine kinase-MB, U/L	8 (5-12)	7 (6-11)	7 (6-10)	9 (6-12)	0.57
Pharmacotherapy, no. (%)					
Quinolones	75 (63%)	80 (69%)	87 (70%)	83 (68%)	0.66
Cephalosporins	52 (44%)	48 (41%)	63 (51%)	69 (57%)	0.08
Ribavirin	105 (88%)	101 (87%)	110 (89%)	100 (82%)	0.39
Oseltamivir	26 (22%)	27 (23%)	20 (16%)	44 (36%)	0.003
Arbidol	35 (29%)	32 (28%)	35 (28%)	25 (20%)	0.39
Glucocorticoid therapy	68 (57%)	64 (55%)	86 (69%)	64 (52%)	0.04
Intravenous immunoglobulin	72 (61%)	64 (55%)	63 (51%)	54 (44%)	0.08
Noninvasive ventilation, no. (%)	13 (11%)	11 (9%)	17 (14%)	35 (29%)	<0.001
Outcomes					
Hospital stays for survivors, median (IQR), d	21 (15-31)	24 (17-31)	27 (17-37)	28 (19-42)	0.008
Hospital mortality, no. (%)	2 (2%)	8 (7%)	9 (7%)	40 (33%)	<0.001

APACHE II: Acute Physiology and Chronic Health Evaluation II; ARDS: acute respiratory distress syndrome; COVID-19: coronavirus disease 2019; FiO2: a fraction of inspired oxygen; IQR: interquartile range; PaO2: partial pressure of oxygen; SOFA: Sequential Organ Failure Assessment.

**Table 2:** Association between cystatin C levels and clinical characters in COVID-19 patients.

Group	Number	Cystatin C levels, mg/L median (IQR)	*P* value
Sex			
Male	227	1.20 (0.98-1.49)	<0.001
Female	254	1.00 (0.84-1.23)	
Age, y			
≥65	173	1.29 (1.05-1.62)	<0.001
<65	308	1.00 (0.84-1.21)	
Comorbidity			
With chronic pulmonary disease	37	1.27 (1.05-1.75)	<0.001
Without chronic pulmonary disease	444	1.07 (0.89-1.33)	
With hypertension	184	1.29 (1.02-1.66)	<0.001
Without hypertension	297	1.01 (0.85-1.23)	
With diabetes	91	1.33 (0.98-1.83)	<0.001
Without diabetes	390	1.06 (0.89-1.30)	
With chronic kidney disease	26	3.22 (1.88-7.40)	<0.001
Without chronic kidney disease	455	1.07 (0.89-1.31)	
With cardiovascular disease	61	1.19 (1.02-1.64)	0.006
Without cardiovascular disease	420	1.07 (0.89-1.33)	
With cerebrovascular disease	42	1.68 (1.30-2.28)	<0.001
Without cerebrovascular disease	439	1.06 (0.89-1.31)	
Level of blood creatinine, *μ*mol/L			
≥97	55	2.08 (1.46-3.48)	<0.001
<97	426	1.05 (0.88-1.27)	
Admission APACHE II score			
≥8	83	1.57 (1.31-2.41)	<0.001
<8	398	1.03 (0.88-1.24)	
Severity of COVID-19			
Critical illness	104	1.49 (1.12-2.27)	<0.001
Noncritical illness	377	1.03 (0.88-1.23)	
Outcome			
Survivors	422	1.05 (0.88-1.29)	<0.001
Nonsurvivors	59	1.62 (1.32-2.41)	

APACHE II: Acute Physiology and Chronic Health Evaluation II; COVID-19: coronavirus disease 2019; IQR: interquartile range.

**Table 3:** Association of cystatin C with mortality and critical illness in all patients with COVID-19.

Cystatin C levels, mg/L	Quartile 1 (<0.90)	Quartile 2 (0.90-1.08)	Quartile 3 (1.08-1.36)	Quartile 4 (≥1.36)	ln-transformed Cystatin C, mg/L∗
No. of death/total	2/119	8/116	9/124	40/122	
Crude model	1 (ref.)	4.33 (0.90, 20.85) 0.0674	4.58 (0.97, 21.64) 0.0549	28.54 (6.71, 121.37) <0.0001	7.04 (3.86, 12.85) <0.0001
Model 1	1 (ref.)	3.55 (0.72, 17.51) 0.1202	2.26 (0.46, 11.06) 0.3147	9.98 (2.24, 44.54) 0.0026	4.23 (2.34, 7.65) <0.0001
Model 2	1 (ref.)	3.56 (0.70, 18.08) 0.1254	2.22 (0.44, 11.21) 0.3325	7.80 (1.64, 37.02) 0.0097	3.62 (1.61, 8.16) 0.0019
Model 3	1 (ref.)	7.66 (0.89, 66.19) 0.0642	4.69 (0.55, 40.01) 0.1580	15.54 (1.90, 126.80) 0.0104	5.64 (1.92, 16.57) 0.0017
No. of critical/total	10/119	12/116	20/124	62/122	
Crude model	1 (ref.)	1.26 (0.52, 3.04) 0.6101	2.10 (0.94, 4.69) 0.0716	11.26 (5.38, 23.57) <0.0001	23.73 (10.66, 52.83) <0.0001
Model 1	1 (ref.)	1.08 (0.44, 2.64) 0.8682	1.33 (0.58, 3.10) 0.5015	5.93 (2.66, 13.21) <0.0001	13.91 (6.11, 31.68) <0.0001
Model 2	1 (ref.)	1.04 (0.42, 2.58) 0.9289	1.33 (0.56, 3.13) 0.5138	3.57 (1.51, 8.46) 0.0039	8.83 (3.52, 22.15) <0.0001
Model 3	1 (ref.)	1.20 (0.47, 3.06) 0.7005	1.41 (0.58, 3.42) 0.4473	2.86 (1.13, 7.28) 0.0272	5.84 (1.91, 17.84) 0.0019

APACHE II: Acute Physiology and Chronic Health Evaluation II; COVID-19: coronavirus disease 2019. Data are shown as odds ratios (OR) and 95% confidence intervals (CI). ∗Modeled as per unit increase of ln-transformed cystatin C. Model 1 adjusted for age and sex. Model 2 adjusted for model 1 plus chronic pulmonary disease, hypertension, diabetes, cardiovascular disease, cerebrovascular disease, and chronic kidney disease. Model 3 adjusted for model 1 plus model 2 plus blood creatinine and APACHE II score.

**Table 4:** Association of cystatin C with mortality and critical illness in COVID-19 patients without chronic kidney disease.

Cystatin C levels, mg/L	Quartile 1 (<0.89)	Quartile 2 (0.89-1.06)	Quartile 3 (1.06-1.31)	Quartile 4 (≥1.31)	ln-transformed Cystatin C, mg/L∗
No. of death/total	2/110	5/110	7/119	33/116	
Crude model	1 (ref.)	2.57 (0.49, 13.55) 0.2652	3.37 (0.69, 16.61) 0.1346	21.47 (5.01, 92.04) <0.0001	17.69 (6.58, 47.52) <0.0001
Model 1	1 (ref.)	2.22 (0.41, 12.01) 0.3560	1.63 (0.32, 8.37) 0.5569	7.67 (1.70, 34.69) 0.0081	5.95 (2.01, 17.63) 0.0013
Model 2	1 (ref.)	2.40 (0.43, 13.56) 0.3204	1.85 (0.35, 9.83) 0.4689	8.40 (1.75, 40.19) 0.0077	5.73 (1.79, 18.32) 0.0032
Model 3	1 (ref.)	5.25 (0.56, 49.57) 0.1474	3.87 (0.43, 34.58) 0.2259	16.77 (1.99, 141.06) 0.0095	6.61 (1.64, 26.70) 0.0080
No. of critical/total	10/110	9/110	16/119	47/116	
Crude model	1 (ref.)	0.89 (0.35, 2.29) 0.8104	1.55 (0.67, 3.59) 0.3022	6.81 (3.22, 14.40) <0.0001	18.78 (7.82, 45.08) <0.0001
Model 1	1 (ref.)	0.79 (0.30, 2.05) 0.6220	0.97 (0.40, 2.34) 0.9518	3.60 (1.59, 8.11) 0.0020	10.07 (3.90, 26.05) <0.0001
Model 2	1 (ref.)	0.77 (0.29, 2.02) 0.5918	1.02 (0.42, 2.47) 0.9704	3.51 (1.51, 8.16) 0.0036	8.89 (3.28, 24.13) <0.0001
Model 3	1 (ref.)	0.88 (0.32, 2.37) 0.7969	1.06 (0.42, 2.66) 0.8987	2.96 (1.20, 7.35) 0.0190	6.37 (2.03, 19.93) 0.0015

APACHE II: Acute Physiology and Chronic Health Evaluation II; COVID-19: coronavirus disease 2019. Data are shown as odds ratios (OR) and 95% confidence intervals (CI). ∗Modeled as per unit increase of ln-transformed cystatin C. Model 1 adjusted for age and sex. Model 2 adjusted for model 1 plus chronic pulmonary disease, hypertension, diabetes, cardiovascular disease, and cerebrovascular disease. Model 3 adjusted for model 1 plus model 2 plus blood creatinine and APACHE II score.

## Data Availability

The datasets generated and/or analyzed in this study are not publicly available because of the respect to and the protection of patient privacy but are available from the corresponding authors on reasonable request.
